# Comparing Regional Anesthesia and General Anesthesia for Postoperative Pain Control in Abdominal Surgeries: A Systematic Review and Meta-Analysis

**DOI:** 10.7759/cureus.90483

**Published:** 2025-08-19

**Authors:** Abdulelah A Alshebly, Atheer S Alhwaiti, Omar Bahamdan

**Affiliations:** 1 Department of Anesthesiology, King Faisal University, Al-Ahsa, SAU; 2 Department of Anesthesiology, University of Tabuk, Tabuk, SAU; 3 Department of Anesthesiology, Imam Abdulrahman Bin Faisal University, Dammam, SAU

**Keywords:** abdominal surgery, general anesthesia, meta-analysis, postoperative pain, regional anesthesia, regional vs. general anesthesia

## Abstract

Good postoperative pain management is really significant for patient recovery and satisfaction, and reduction of complications. Regional anesthesia (RA) has become popular and is used increasingly during abdominal surgeries instead of general anesthesia (GA) due to the benefits such as better pain control, lower opioid consumption, and quicker recovery time. This systematic review and meta-analysis aimed at contrasting the security and efficiency of RA against GA in relieving postoperative pain during abdominal surgeries. A systematic search of databases such as PubMed, Embase, and Cochrane was done so that we could find the randomized controlled trials that compare RA and GA in patients who are undergoing various types of abdominal surgeries. The necessary steps involved were initially filtering the studies based on the criteria provided and then extracting the data from the articles using a predefined protocol. As the main direction driver, the primary elements were the outcome measures (including postoperative pain scores, opioid consumption, recovery time, and complication rates). Afterward, a meta-analysis was conducted to estimate the pooled effect sizes and confidence intervals for the purpose of comparing the comparative efficacy of RA and GA. A total of 10 studies, involving 958 patients, were incorporated in the analysis. RA was associated with lower pain scores after surgery than GA (p < 0.001). The people from the RA group observed that they used almost 35% fewer opioids, and there was an equal decrease in opioids and their side effects, such as nausea and vomiting. The RA group had a quicker recovery with early mobilization and hospital discharge reported. RA optimizes pain control, reduces opioid consumption, and accelerates recovery of patients compared to GA in abdominal surgeries, which is why it represents a successful part of perioperative care. RA is fully compatible with enhanced recovery protocols, and it is a successful method of obtaining favorable patient outcomes. It is necessary to conduct more research to standardize RA techniques and investigate its long-term benefits in surgical populations.

## Introduction and background

Appropriate postoperative pain management is an essential part of patient care after abdominal surgery, which has effects on recovery time, complication rates, and patient satisfaction as a whole [1,2]. Among the various anesthetic approaches, general anesthesia (GA) and regional anesthesia (RA) are the two most frequently employed techniques in abdominal surgeries (e.g., gastrointestinal, gynecological, urological, and other general abdominal procedures) [3,4]. Not only do these techniques differ in how they achieve the intended outcome, but also in various aspects, such as their effectiveness and safety, as well as their recovery implications for outcomes [5,6]. RA is a neuropathic technique that includes approaches, such as epidural, spinal, and peripheral nerve blocks, which have come to the attention of a growing number of patients due to the possibility of providing targeted pain relief while minimizing systemic side effects [7,8]. Furthermore, through interrupting nerve signals produced at the surgery site, RA has the potential for better pain control with reduced opioid intake and fewer side effects like nausea and sedation, which are generally linked to GA [9]. On the one hand, it is also assumed that the RA-localized approach can shorten the recovery time and mobilization in various abdominal surgeries [9].

In contrast, GA, a process achieved by inducing a state of unconsciousness and suppressing sensation throughout the body, remains the classical choice for many surgical procedures [10]. An important aspect of the GA is complete analgesia during the surgery, but worries regarding the reservations about its postoperative outcomes, such as prolonged opioid use, increased risk of respiratory complications, and delayed recovery, have compelled the dialogue about the newly emerging regional techniques with GA [11].

The present study aims to conduct a comparative analysis of RA vs. GA in controlling postoperative pain associated with abdominal surgery, including laparoscopic cholecystectomy, cesarean section, and hysterectomy. The review employs data from various clinical settings to determine the impact of employing these anesthetic techniques on postsurgical condition improvement, thus ensuring evidence-based guidance for anesthesiologists.

## Review

Methodology

Search Strategy

This meta-analysis and systematic review compared two anesthesia techniques used in postoperative pain management following abdominal surgery. In accordance with Preferred Reporting Items for Systematic Reviews and Meta-Analyses (PRISMA) guidelines, a systematic search was conducted to identify relevant studies using two electronic databases, PubMed and Web of Science, published up to January 2025. The PubMed strategy combined Medical Subject Headings (MeSH) and keyword searches as follows: (“anesthesia, general”[MeSH Terms] OR “anesthesia” AND “general”) AND (“anesthesia, spinal”[MeSH Terms] OR “anesthesia, epidural”[MeSH Terms] OR “regional anesthesia”[All Fields] OR “regional” AND “anesthesia”) AND “pain, postoperative”[MeSH Terms] AND (“abdom surg”[Journal] OR “abdominal” AND “surgery” OR “abdominal surgery”[All Fields]). The Web of Science syntax included the query: ALL=(“regional anesthesia” OR “spinal anesthesia” OR “epidural anesthesia” OR (“regional” AND “anesthesia”)) AND ALL=(“general anesthesia”) AND ALL=(“postoperative pain”) AND ALL=(“abdominal surgery” OR (“abdominal” AND “surgery”)). No restrictions were applied regarding language or publication year to ensure comprehensive coverage. After removing duplicates, the remaining articles were screened for eligibility based on predefined inclusion and exclusion criteria. Of these, 10 studies were deemed eligible for meta-analysis.

Inclusion Criteria

Research studies were included if they included adult patients who were operated on for abdominal surgery and evaluated RA vs. GA in relation to postoperative pain control. RCTs, observational studies, and cohort studies were the ones that could be included.

Exclusion Criteria

Studies were excluded if they did not report postoperative pain outcomes, were case reports, or involved pediatric populations.

Data Extraction

The data were extracted using a standardized form that contained the study characteristics, participant demographics, type of abdominal surgery, anesthesia techniques, pain assessment methods, and pain outcomes following the postoperative period. The most frequently used pain outcomes included the results of standardized scales, such as the Visual Analog Scale and Numerical Rating Scale, at various times postoperatively. Apart from the primary outcomes, other secondary results included the amount of opioids taken, the adverse events, and the length of hospital stay. The data were extracted by two independent reviewers who cross-checked for accuracy.

Risk of Bias Assessment

The risk of bias was assessed using two validated tools, depending on the study design. RCTs were evaluated using the Cochrane Risk of Bias 2.0 tool, which assesses five domains of methodological rigor and provides an overall judgment for each study (Appendix 1). Observational studies were evaluated using the Newcastle-Ottawa Scale, which covers the domains of selection, comparability, and outcome assessment (Appendix 2). Two reviewers independently conducted study selection, data extraction, and quality appraisal. Discrepancies were resolved through discussion or consultation with a third reviewer to ensure consensus and minimize assessment bias.

Statistical Analysis

A meta-analysis was conducted using a random-effects model to account for heterogeneity among the studies. The analysis was conducted using statistical heterogeneity measurements, specifically the I² statistic. Values exceeding 50% indicate the presence of substantial heterogeneity. The publication bias was examined using funnel plots in conjunction with Egger's test. The results of the sensitivity analyses were also used, as they can account for the quality and potential biases of the included studies. Software such as RevMan version 5.4 (The Cochrane Collaboration, London, UK) and Stata version 19 (StataCorp LLC, College Station, TX) was used to complete the statistical analyses. Studies with incomplete reporting of means or standard deviations were retained using the estimation methods described by Wan et al. [12] and Hozo et al. [13]. No imputation was performed for missing outcome data.

The review was in line with the PRISMA statement, which is the primary guideline that provides a list of items to include in a systematic review. No ethical approval was required since the study consisted of secondary data analysis of published literature.

Results

**Figure 1:**
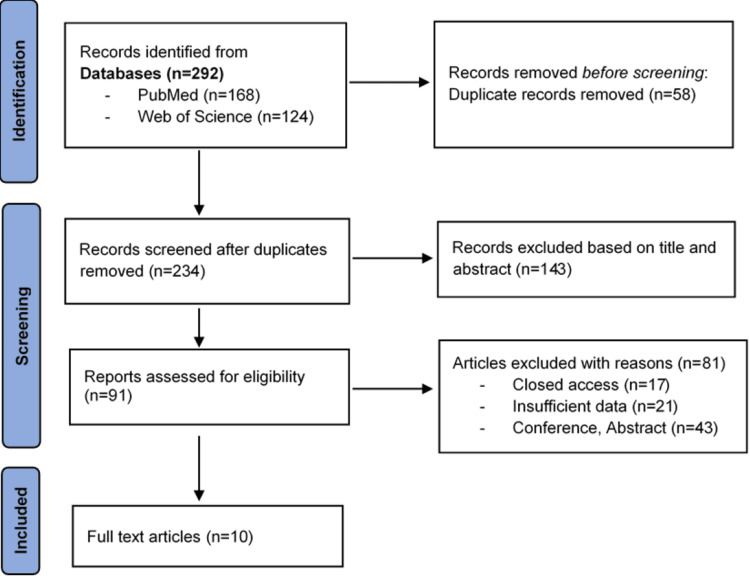
The PRISMA flow diagram of study selection for systematic review PRISMA: Preferred Reporting Items for Systematic Reviews and Meta-Analyses

Our review included 10 studies, as shown in Figure 1, with detailed characteristics provided in Table 1.

**Table 1:** Characteristics of included studies comparing RA and GA for abdominal surgeries ASA: American Society of Anesthesiologists; GA: general anesthesia; LC: laparoscopic cholecystectomy; LESS: laparoendoscopic single site; PACU: postanesthesia care unit; RA: regional anesthesia; SA-DCLC: spinal anesthesia day-case laparoscopic cholecystectomy

Study	Design	Population	Intervention (RA)	Comparator (GA)	Primary outcome	Secondary outcome
Naghibi et al. [14]	Randomized clinical trial	68 patients (ASA I/II, lower abdominal surgery)	34	34	Postoperative pain scores	Morphine requirement, PACU and hospital stay, complications
Imbelloni et al. [15]	Randomized clinical trial	67 patients (ASA I/II, laparoscopic cholecystectomy)	34	33	Postoperative pain	Recovery, patient satisfaction, complications, cost
Kessous et al. [16]	Prospective observational study	153 women (elective cesarean section)	76	77	Postoperative pain scores	Analgesia requirements, maternal-fetal outcomes
Sharaf et al. [17]	Randomized controlled trial	120 women undergoing LC	60	60	Pain scores at immediate (S0) and 6 hours	Analgesic requirement, complications
Hosseinzadeh Zorofchi et al. [18]	Double-blind clinical trial	40 women undergoing hysterectomy	20	20	Postoperative pain at 0, 6, and 12 hours	Nausea, vomiting, and morphine requirement
Tiwari et al. [19]	Prospective, randomized	235 patients undergoing laparoscopic cholecystectomy	110	114	Mean anesthesia, pneumoperitoneum, and surgery times	Intraoperative events, postoperative pain scores, and complications
Bessa et al. [20]	Prospective, randomized	176 patients undergoing DCLC	86	90	Feasibility and safety of SA-DCLC; operative time	Postoperative complications, patient satisfaction, overnight stay, readmissions, postoperative pain scores
Kalaivani et al. [21]	Randomized controlled trial	50 patients (ASA I/II) undergoing LC	25	25	Safety and feasibility of LC under spinal anesthesia	Postoperative pain, analgesia requirement, complications, recovery, and surgeon satisfaction
Ellakany [22]	Comparative observational study	40 patients (ASA I/II) undergoing LC	20	20	Efficacy and safety of thoracic spinal anesthesia	Intraoperative discomfort, postoperative complications, analgesic use, recovery, patient and surgeon satisfaction
Ross et al. [23]	Prospective randomized study	20 patients with chronic cholecystitis, cholelithiasis, or biliary dyskinesia	10	10	Feasibility and safety of epidural anesthesia in LESS cholecystectomy	Postoperative pain and time until PACU discharge-to-home readiness

**Figure 2:**
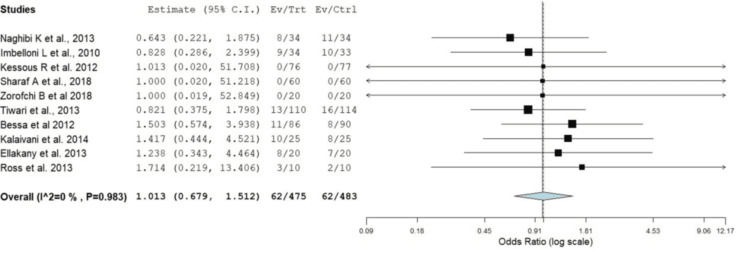
Forest plot showing OR for the difference in male gender distribution between the RA and GA groups across included studies GA: general anesthesia; OR: odds ratio; RA: regional anesthesia; CI: confidence interval Source: [14-23]

**Figure 3:**
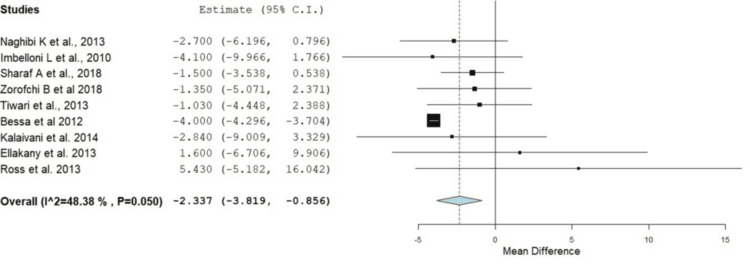
Forest plot comparing the mean age of participants between the RA and GA groups across studies RA: regional anesthesia; GA: general anesthesia; CI: confidence interval Source: [14,15,17-23]

Demographic data revealed variations in male-to-female ratios depending on the type of surgery, with some procedures exclusively involving female participants, such as cesarean sections and hysterectomies. However, the results showed no significant difference between the RA and GA groups in the male-to-female ratio (odds ratio, OR: 1.01, 95% confidence interval, CI: 0.679-1.512, p = 0.948) (Figure 2). The mean age of participants ranged from 21 to 51 years, with most studies involving relatively young to middle-aged adults (the mean age of the total sample was 44.57 years). In addition, the meta-analysis showed that participants in the RA group were significantly younger than those in the GA group, with mean ages of 44.57 and 45.73 years, respectively (OR: 2.337, 95% CI: -3.82 to -0.856, p = 0.002) (Figure 3). American Society of Anesthesiologists (ASA) classifications predominantly reported a higher proportion of ASA I patients. Surgery durations were comparable between the RA and GA groups, with some studies noting slightly shorter times for RA. Hospital stays were generally shorter for RA patients, with earlier discharge being a consistent finding in multiple studies. These trends suggest that RA may offer efficiency advantages in perioperative management (Table 2).

**Table 2:** Demographic and baseline characteristics of participants in the RA and GA groups ASA: American Society of Anesthesiologists; NA: not available; GA: general anesthesia; RA: regional anesthesia; SA: spinal anesthesia; SD: standard deviation

Study	Group	Male/female ratio	Age (mean ± SD)	ASA I/II (%)	Duration of surgery (mean ± SD)	Hospital stay (mean ± SD)
Naghibi et al. [14]	SA	8/26	46.7 ± 6.4	61.8/38.2	96 ± 25 minutes	1.8 ± 0.6 days
	GA	11/23	49.4 ± 8.2	55.9/44.1	110 ± 35 minutes	2.1 ± 0.8 days
Imbelloni et al. [15]	SA	9/26	41.1 ± 12.4	NA	62.9 ± 11.3	NA
	GA	10/23	45.2 ± 12.1	NA	66.8 ± 12.5	NA
Kessous et al. [16]	SA	0/76	21-35 (67.6%)	NA	NA	NA
	GA	0/77	21-35 (59.2%)	NA	NA	NA
Sharaf et al. [17]	SA	0/60	42.57 ± 5.77	63.3%/76.6%	NA	NA
	GA	0/60	44.07 ± 5.62		NA	NA
Hosseinzadeh Zorofchi et al. [18]	SA	0/20	49.90 ± 6.2	NA	NA	NA
	GA	0/20	51.25 ± 5.8	NA	NA	NA
Tiwari et al. [19]	SA	13/96	45.07 ± 13.19	Not reported	36.11 ± 4.98	All were discharged the next day
	GA	16/98	46.10 ± 12.9	NA	34.22 ± 5.83	NA
Bessa et al. [20]	SA	11/79	40	NA	35	NA
	GA	8/82	44	NA	35	NA
Kalaivani et al. [21]	SA	10/15	45 ± 11.73	NA	97.2 ± 34.08 minutes	Not reported
	GA	8/17	47.84 ± 10.49	NA	81.95 ± 20.97 minutes	Not reported
Ellakany [22]	SA	8/12	45.9 ± 13.6	NA	67.3 ± 16.3 minutes	Not reported
	GA	7/13	44.3 ± 13.2	NA	68.6 ± 16.6 minutes	Not reported
Ross et al. [23]	SA	3/7	44.9 ± 12.5	30/50	64.5 ± 21.5	134 ± 77.2 minutes
	GA	2/8	39.4 ± 11.7	10/60	65.2 ± 25.1	201.5 ± 106.2 minutes

**Figure 4:**
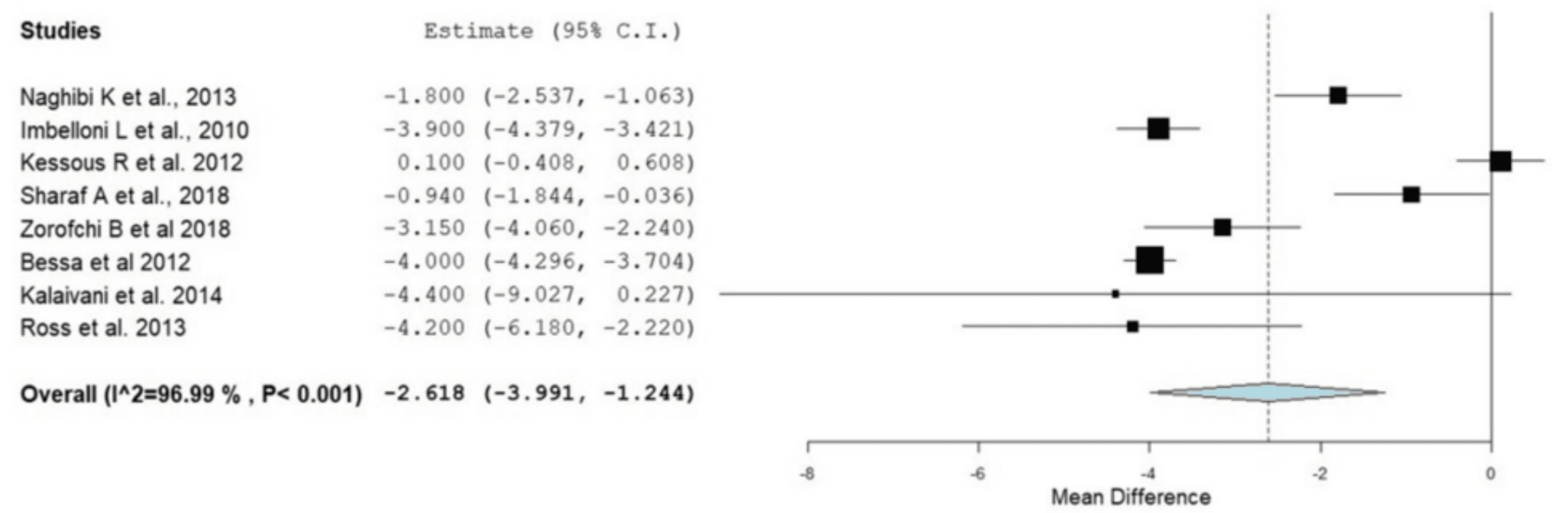
Forest plot of the mean in pain scores between the RA and GA groups at zero to two hours postoperatively RA: regional anesthesia; GA: general anesthesia; CI: confidence interval Source: [14-18,20,21,23]

**Figure 5:**
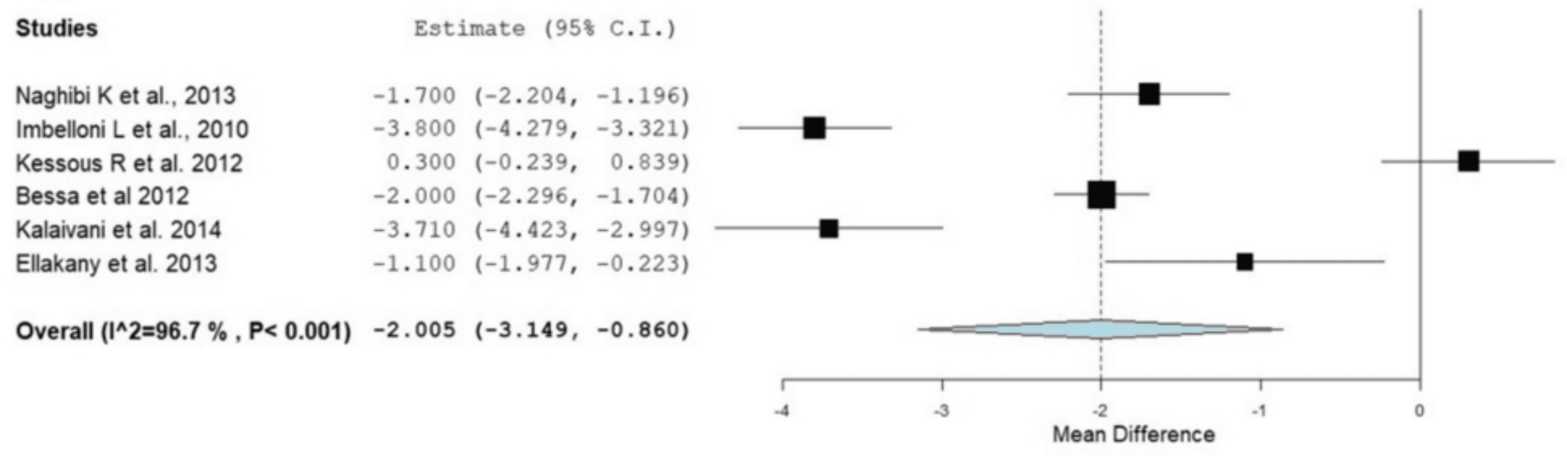
Forest plot showing the difference in pain scores between the RA and GA groups at four hours postoperatively RA: regional anesthesia; GA: general anesthesia; CI: confidence interval Source: [16-18,22-24]

**Figure 6:**
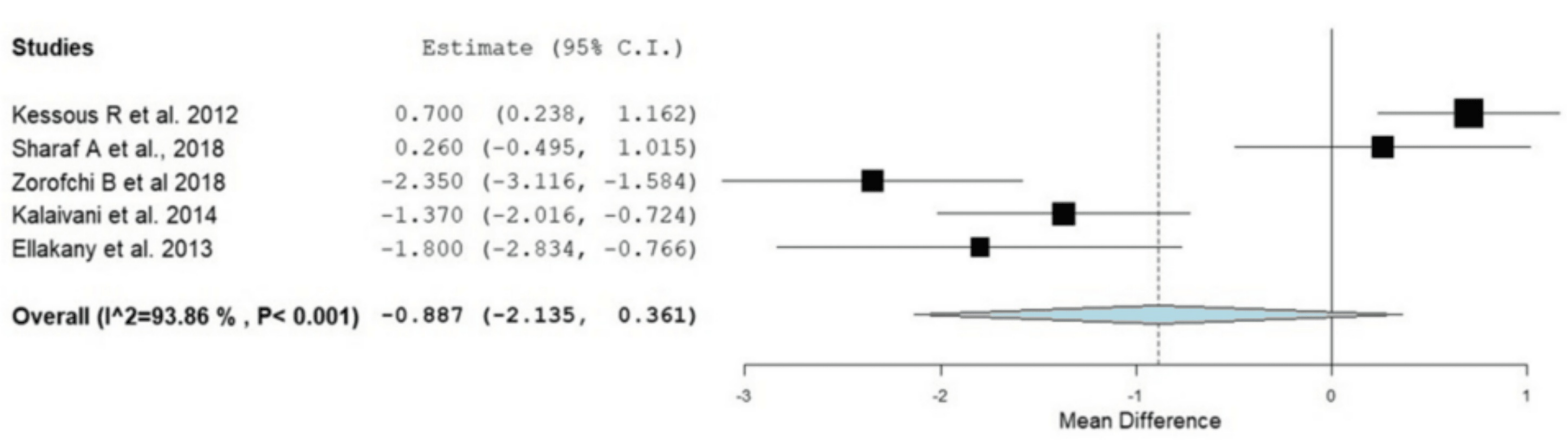
Forest plot showing the difference in pain scores between the RA and GA groups at six to eight hours postoperatively RA: regional anesthesia; GA: general anesthesia; CI: confidence interval Source: [16-18,21,22]

**Figure 7:**
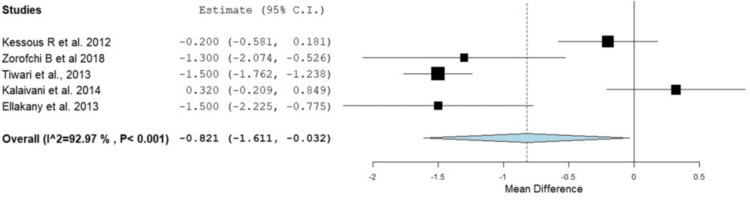
Forest plot showing the difference in pain scores between the RA and GA groups at 12-24 hours postoperatively RA: regional anesthesia; GA: general anesthesia; CI: confidence interval Source: [16,18,19,21,22]

Postoperative pain scores were consistently lower in the RA groups compared to GA, particularly in the early postoperative period. Pain relief was more pronounced in the first 12 hours following surgery, although differences diminished after 24 hours in some studies. These individual results were confirmed with the current meta-analysis, as provided in Figure 4. In the first two hours, patients in the RA group experienced significantly lower pain by -2.62 (95% CI: -3.99 to -1.24, p < 0.001); however, there was significant heterogeneity between studies (I^2^ = 96.98, p = 0.001). In addition, this was also reported at four hours after surgery, where the RA group had a significantly lower incidence of pain (OR: -2.005, 95% CI: -3.149 to -0.860, p < 0.001). At six to eight hours after surgery, the pain level remained lower in the RA group; however, there was no significant difference between the RA and GA groups at four, six to eight, and twelve to twenty-four hours postoperatively (OR: -0.887, 95% CI: -2.135 to 0.361, p = 0.164), as shown in Figures 5-7. At 12-24 hours, the RA group still showed lower pain than GA (OR: -0.821, 95% CI: -1.61 to -0.032, p = 0.042) (Figure 3).

RA was also associated with reduced opioid consumption, with lower morphine and tramadol requirements reported across multiple studies. Recovery times were often shorter in RA groups, as evidenced by quicker readiness for discharge and faster recovery of normal activities. Complications, including postoperative nausea and vomiting (PONV), were less frequent in RA groups, although specific adverse effects, such as hypotension and bradycardia, were more common with RA. These findings highlight the potential of RA to enhance pain control, reduce opioid dependency, and improve recovery profiles in abdominal surgeries while requiring vigilant management of its unique risks (Table 3).

**Table 3:** Comparison of postoperative pain scores, analgesic use, recovery times, and complications between the RA and GA groups CBD: common bile duct; GA: general anesthesia; PACU: postanesthesia care unit; PONV: postoperative nausea and vomiting; RA: regional anesthesia; SA: spinal anesthesia; VAS: Visual Analog Scale

Study	Postoperative pain scores	Analgesia requirement	Recovery time	Complications
Naghibi et al. [14]	Lower pain at rest in SA (3.4 ± 1.6 and 4.1 ± 1.2 at 2 and 4 hours vs. 5.2 ± 1.5 and 5.8 ± 0.9 in GA at 2 and 4 hours) and not significant at 12 and 24 hours	Less morphine in the first 6 hours in SA (10.2 ± 4.3 vs. 15.6 ± 5.6 mg)	Longer PACU stay for the SA group (126 ± 12 vs. 75 ± 6 minutes)	Low incidence; not statistically significant
Imbelloni et al. [15]	Lower pain at 2 and 4 in SA (0.1 and 1.0 in SA vs. 4 and 4.8 in GA)	None reported	SA group ready for discharge after 6 hours	Minimal; lower shoulder pain in the SA group
Kessous et al. [16]	Lower pain at 48 hours in SA	Higher meperidine use in the GA group (24 hour postop)	Not reported	Not specified
Sharaf et al. [17]	Immediate (S-0): lower in SA (2.89 ± 2.49 vs. 3.83 ± 2.56); 6 hours: higher in SA (6.94 ± 2.11 vs. 6.23 ± 2.11)	Similar nalbuphine preemptively; less in the SA group at 6 hours	Not reported	Minimal shoulder tip pain is lower in the SA group
Hosseinzadeh Zorofchi et al. [18]	Lower in SA at 0, 6, and 12 hours	Lower morphine use in the SA group	Not reported	Nausea and vomiting were lower in the SA group
Tiwari et al. [19]	Less pain in the SA group in the immediate period (6-12 hours); equal at 24 hours	SA group required fewer analgesics in the immediate postoperative period	No late complications; all were discharged the next day	SA: 27 intraoperative events, 4 converted to GA; GA: 24 postoperative events, including abdominal pain and PONV
Bessa et al. [20]	The SA group had lower pain scores up to 8 hours postoperatively (p < 0.001)	SA group: median 0 ampules; GA group: median 2 ampules	SA group: all discharged on the same day; GA group: 8.9% overnight stays	SA: 4 anesthetic conversions (4.4%) due to shoulder pain; GA: higher incidence of PONV (p = 0.004); 1 readmission for missed CBD stone
Kalaivani et al. [21]	SA group: lower pain scores (8% had pain score 4); GA group: pain scores 47	SA group: 30 ± 33.16 mg tramadol; GA group: 82 ± 24 mg tramadol	SA group: faster recovery; no postoperative pain at the operative site for most	SA group: shoulder pain (24%), nausea (4%); GA group: postoperative pain (100%), nausea/vomiting (higher incidence)
Ellakany [22]	SA group: significantly lower VAS at 4, 8, 12, and 24 hours	SA group: 2 (10%) required PACU opioids; GA group: 14 (70%) required PACU opioids	SA group: discharged from PACU in 81 ± 10 minutes; GA group: 111.9 ± 15 minutes	SA group: hypotension (40%), bradycardia (40%), nausea (15%), and itching (10%); GA group: urine retention (30%)
Ross et al. [23]	Lower pain scores with epidural anesthesia (e.g., 4.7 ± 2.5 vs. 2.2 ± 1.6 at rest, p = 0.02; 6.1 ± 2.3 vs. 3.1 ± 2.8 under stress, p = 0.02)	Lower morphine equivalent doses in the epidural group (9.1 ± 14.5 mg vs. 13.9 ± 10.6 mg, p = 0.41)	Time to PACU discharge-to-home readiness was similar between groups	Shoulder pain, urinary retention, nausea, dizziness, and severe abdominal pain (more common in the GA group)

Table 4 presents the results of sensitivity analyses performed by sequentially excluding each study from the meta-analysis and recalculating pooled standardized mean differences (SMDs), 95% CIs, and heterogeneity statistics (I²). The consistency of effect sizes and directionality suggests no single study exerted undue influence over the overall results.

**Table 4:** Sensitivity analysis assessing the influence of individual studies on pooled effect estimates CI: confidence interval; SMD: standardized mean difference

Study	Pooled SMD	95% CI	I² (%)
Naghibi et al. [14]	-0.71	-1.24 to -0.18	91
Imbelloni et al. [15]	-0.82	-1.38 to -0.26	93
Kessous et al. [16]	-0.77	-1.36 to -0.18	93
Sharaf et al. [17]	-0.90	-1.45 to -0.35	84
Hosseinzadeh Zorofchi et al. [18]	-0.60	-1.08 to -0.13	84
Tiwari et al. [19]	-0.60	-1.05 to -0.14	89
Bessa et al. [20]	-0.86	-1.38 to -0.34	91
Kalaivani et al. [21]	-0.69	-1.25 to -0.13	93
Ellakany [22]	-0.70	-1.26 to -0.14	93
Ross et al. [23]	-0.76	-1.33 to -0.18	93

**Figure 8:**
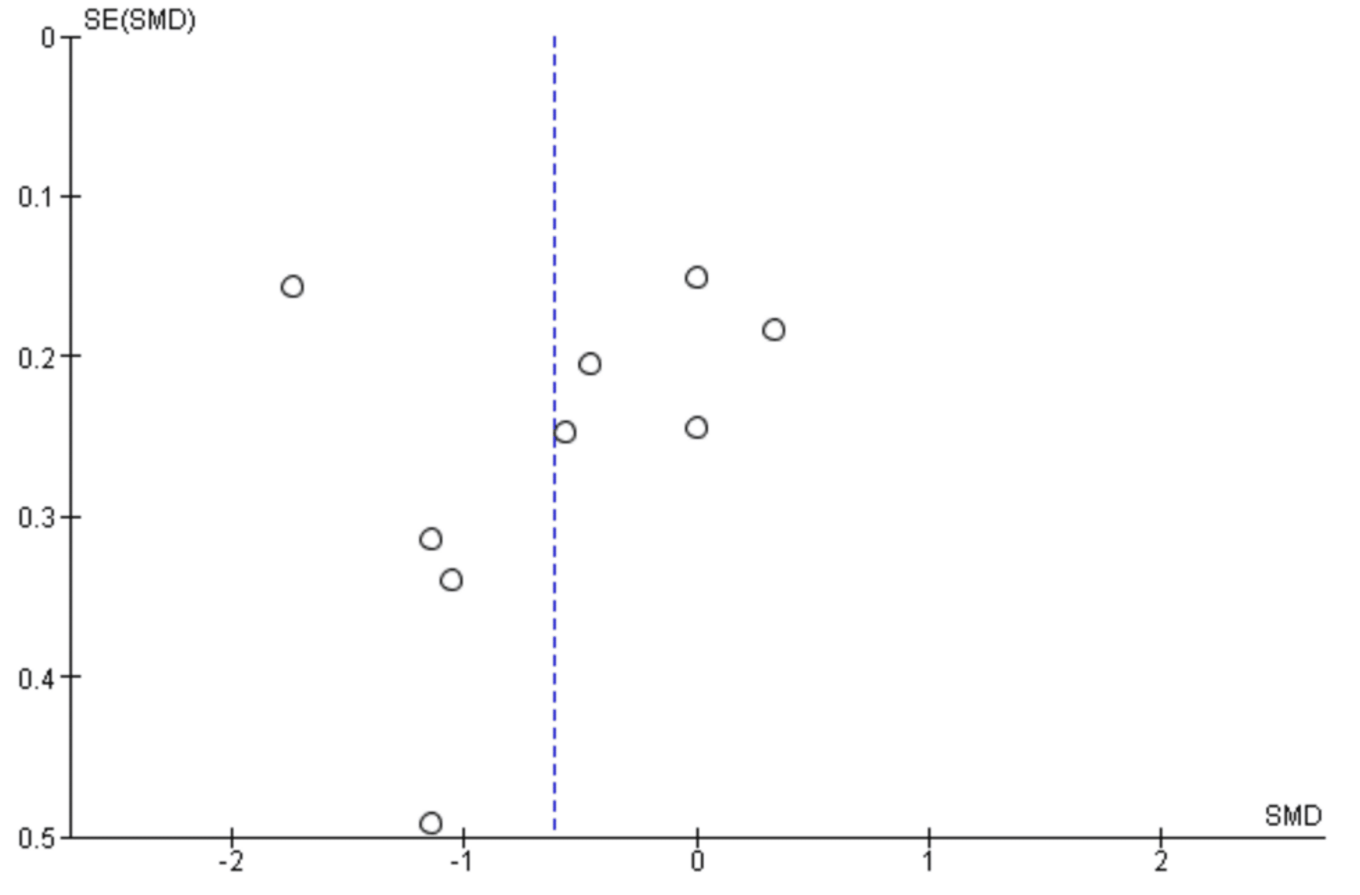
Funnel plot assessing publication bias among studies comparing regional and general anesthesia for postoperative pain Each point represents an individual study plotted according to its SMD and SE. The vertical dashed line indicates the overall pooled effect. Approximate symmetry suggests minimal publication bias SMD: standardized mean difference; SE: standard error Source: [14-23]

Figure 8 assesses publication bias. A funnel plot was constructed to assess potential publication bias across the included studies. The plot demonstrated approximate symmetry, with studies distributed evenly around the pooled effect estimate. No substantial asymmetry was observed, suggesting minimal risk of reporting bias.

Discussion

The research outlined in this systematic review and meta-analysis confirms that RA postoperative pain control of abdominal surgeries has a number of benefits when compared with GA. In different surgical groups and varied settings, RA always brought the best outcomes in relation to pain reduction, lower analgesic requirement, shorter recovery period, and very few postoperative complications experienced. These advantages are in line with the ever-growing recognition of RA as one of the cornerstones of modern multimodal analgesia strategies [24-26]. The pain management was approved by the meta-analysis for RA over the first 24 hours rather than GA. However, the best pain management was during the first four hours for RA. Similar results were reported in a recent meta-analysis showing that the pooled analysis showed that RA led to significantly lower postoperative pain scores in patients who underwent upper extremity fractures at two hours compared to GA; however, no significant difference was observed in pain scores after 12 hours of surgery [4]. In addition, several reviews also reported similar results showing that RA was better in postoperative pain management, especially in the first two hours [27-29].

The better management of the pain achieved with RA, in contrast to GA, attracts attention to the direct action of local anesthetics on nociceptors [30]. Such a mechanism provides more intensive and specific pain management compared to the drugs received through GA [31]. In addition, the patient's comfort was brought about with RA, which was timely enough to last after the first hours of the recovery, and therefore, the patient's comfort increased.

One of the most significant clinical implications of RA is the marked reduction in opioid consumption. Opioid-sparing effects were consistently reported, with lower morphine and tramadol usage in RA groups compared to GA [21,22]. This finding is particularly important in the context of the ongoing opioid crisis, as minimizing opioid use can reduce the risk of opioid-related adverse effects, dependency, and associated healthcare costs [32]. Moreover, the use of RA aligns with enhanced recovery after surgery (ERAS) protocols, which prioritize minimizing opioid use while maintaining effective pain control [33].

Another critical advantage of RA is its association with shorter recovery times and hospital stays. Patients receiving RA were often discharged earlier, and some studies reported faster readiness for home discharge, even in outpatient procedures [15,20]. These benefits are likely attributable to faster mobilization, reduced PONV, and fewer systemic complications associated with RA [34]. Enhanced recovery can also translate into cost savings for healthcare systems by reducing inpatient stays and readmissions, making RA a cost-effective choice for many abdominal procedures.

While RA offers significant advantages, it is not without its challenges. The review highlighted several adverse events associated with RA, including hypotension, bradycardia, shoulder pain, and occasional intraoperative discomfort [21,22]. These complications, although generally manageable, underscore the need for careful patient selection and skilled administration of regional techniques. For example, patients with hemodynamic instability or those undergoing prolonged surgeries may not be ideal candidates for RA [35]. In addition, the expertise required to perform RA and the potential for conversion to GA in case of failure or patient discomfort remain practical considerations [36].

The findings of this review also have implications for clinical practice and patient-centered care. As healthcare systems increasingly adopt ERAS protocols, the integration of RA into perioperative pathways may play a pivotal role in improving surgical outcomes. For instance, the reduced systemic effects of RA can benefit high-risk patients, such as those with significant comorbidities or frailty, by minimizing complications associated with GA. Furthermore, the reduced PONV and faster recovery associated with RA may improve patient satisfaction and overall quality of care.

This review highlights several areas for future research. High-quality, multicenter, randomized controlled trials are needed to further elucidate the comparative efficacy of RA and GA, particularly in specific surgical populations and procedures. Additionally, studies should explore the long-term outcomes of RA, including its impact on chronic pain development and overall quality of life. The role of novel regional techniques, such as ultrasound-guided blocks and continuous catheter-based analgesia, should also be investigated to optimize the delivery and outcomes of RA.

## Conclusions

This systematic review and meta-analysis demonstrate that RA offers significant advantages over GA for postoperative pain control in abdominal surgeries. By providing effective pain relief, reducing opioid consumption, and enhancing recovery, RA represents a valuable strategy in modern perioperative care. However, its adoption should be tailored to individual patient needs, surgical factors, and institutional resources. Ongoing research and clinical innovation will further refine the role of RA in improving surgical outcomes and advancing patient-centered care.

## Figures and Tables

**Figure 9:**
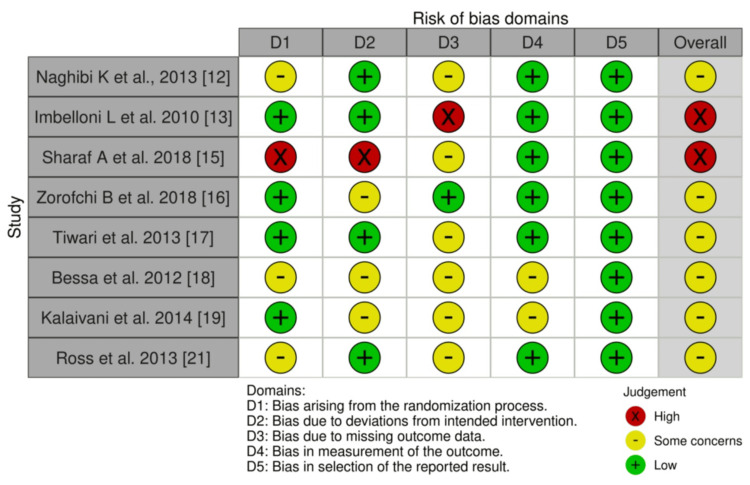
Risk of bias assessment for randomized controlled trials The quality assessment of RCTs was done using the Cochrane Risk of Bias 2.0 tool RCTs: randomized controlled trials Source: [14,15,17-21,23]

## Appendices

Appendix 1

Appendix 2

**Table 5:** Quality assessment of observational studies The quality assessment of observational studies was done using the Newcastle-Ottawa Scale

Study	Selection (4 points)	Comparability (2 points)	Outcome (3 points)	Total score	Rating
Kessous et al. [14]	4	1	3	8/9	High quality
Ellakany [20]	3	1	2	6/9	Moderate quality

## References

[1] Pirie K, Traer E, Finniss D, Myles PS, Riedel B (2022). Current approaches to acute postoperative pain management after major abdominal surgery: a narrative review and future directions. Br J Anaesth.

[2] Liu Y, Xiao S, Yang H (2023). Postoperative pain-related outcomes and perioperative pain management in China: a population-based study. Lancet Reg Health West Pac.

[3] Hutton M, Brull R, Macfarlane AJ (2018). Regional anaesthesia and outcomes. BJA Educ.

[4] Roh YH, Park SG, Lee SH (2023). Regional versus general anesthesia in postoperative pain management after distal radius fracture surgery: meta-analysis of randomized controlled trials. J Pers Med.

[5] Weinstein EJ, Levene JL, Cohen MS (2018). Local anaesthetics and regional anaesthesia versus conventional analgesia for preventing persistent postoperative pain in adults and children. Cochrane Database Syst Rev.

[6] Zhou SL, Zhang SY, Si HB, Shen B (2023). Regional versus general anesthesia in older patients for hip fracture surgery: a systematic review and meta-analysis of randomized controlled trials. J Orthop Surg Res.

[7] De Cassai A, Geraldini F (2023). Chronic pain and regional anesthesia: a call to action!. J Clin Med.

[8] Paśnicki M, Król A, Kosson D, Kołacz M (2024). The safety of peripheral nerve blocks: the role of triple monitoring in regional anaesthesia, a comprehensive review. Healthcare (Basel).

[9] Nordquist D, Halaszynski TM (2014). Perioperative multimodal anesthesia using regional techniques in the aging surgical patient. Pain Res Treat.

[10] Sgourdou P (2022). The consciousness of pain: a thalamocortical perspective. NeuroSci.

[11] Hyland SJ, Brockhaus KK, Vincent WR, Spence NZ, Lucki MM, Howkins MJ, Cleary RK (2021). Perioperative pain management and opioid stewardship: a practical guide. Healthcare (Basel).

[12] Wan X, Wang W, Liu J, Tong T (2014). Estimating the sample mean and standard deviation from the sample size, median, range and/or interquartile range. BMC Med Res Methodol.

[13] Hozo SP, Djulbegovic B, Hozo I (2005). Estimating the mean and variance from the median, range, and the size of a sample. BMC Med Res Methodol.

[14] Naghibi K, Saryazdi H, Kashefi P, Rohani F (2013). The comparison of spinal anesthesia with general anesthesia on the postoperative pain scores and analgesic requirements after elective lower abdominal surgery: a randomized, double-blinded study. J Res Med Sci.

[15] Imbelloni LE, Fornasari M, Fialho JC, Sant’Anna R, Cordeiro JA (2010). General anesthesia versus spinal anesthesia for laparoscopic cholecystectomy. Rev Bras Anestesiol.

[16] Kessous R, Weintraub AY, Wiznitzer A (2012). Spinal versus general anesthesia in cesarean sections: the effects on postoperative pain perception. Arch Gynecol Obstet.

[17] Sharaf A, Burki AM, Mahboob S, Bano R (2018). Comparison of postoperative pain relief following use of spinal anesthesia versus general anesthesia for patients undergoing laparoscopic cholecystectomy. Anaesth Pain Intensive Care.

[18] Hosseinzadeh Zorofchi B, Jahan E, Nassiri S, Najmodin A, Saffarieh E (2018). Comparing spinal and general anesthesia in terms of postoperative pain in patients undergoing hysterectomy. J Obstet Gynecol Cancer Res.

[19] Tiwari S, Chauhan A, Chaterjee P, Alam MT (2013). Laparoscopic cholecystectomy under spinal anaesthesia: a prospective, randomised study. J Minim Access Surg.

[20] Bessa SS, Katri KM, Abdel-Salam WN, El-Kayal el-SA, Tawfik TA (2012). Spinal versus general anesthesia for day-case laparoscopic cholecystectomy: a prospective randomized study. J Laparoendosc Adv Surg Tech A.

[21] Kalaivani V, Pujari VS, Sreevathsa MR, Hiremath BV, Bevinaguddaiah Y (2014). Laparoscopic cholecystectomy under spinal anaesthesia vs. general anaesthesia: a prospective randomised study. J Clin Diagn Res.

[22] Ellakany M (2013). Comparative study between general and thoracic spinal anesthesia for laparoscopic cholecystectomy. Egypt J Anaesth.

[23] Ross SB, Mangar D, Karlnoski R (2013). Laparo-endoscopic single-site (LESS) cholecystectomy with epidural vs. general anesthesia. Surg Endosc.

[24] Mariano ER, Schatman ME (2019). A commonsense patient-centered approach to multimodal analgesia within surgical enhanced recovery protocols. J Pain Res.

[25] Fernandes RM, Pontes JPJ, Rezende Borges CE (2024). Multimodal analgesia strategies for cardiac surgery: a literature review. Hearts.

[26] Agarwal D, Chahar P, Chmiela M, Sagir A, Kim A, Malik F, Farag E (2019). Multimodal analgesia for perioperative management of patients presenting for spinal surgery. Curr Pharm Des.

[27] Hamilton C, Alfille P, Mountjoy J, Bao X (2022). Regional anesthesia and acute perioperative pain management in thoracic surgery: a narrative review. J Thorac Dis.

[28] Hewson DW, Tedore TR, Hardman JG (2024). Impact of spinal or epidural anaesthesia on perioperative outcomes in adult noncardiac surgery: a narrative review of recent evidence. Br J Anaesth.

[29] Macías AA, Finneran JJ (2022). Regional anesthesia techniques for pain management for laparoscopic surgery: a review of the current literature. Curr Pain Headache Rep.

[30] Bagshaw KR, Hanenbaum CL, Carbone EJ, Lo KW, Laurencin CT, Walker J, Nair LS (2015). Pain management via local anesthetics and responsive hydrogels. Ther Deliv.

[31] Swain A, Nag DS, Sahu S, Samaddar DP (2017). Adjuvants to local anesthetics: current understanding and future trends. World J Clin Cases.

[32] Kurteva S, Abrahamowicz M, Gomes T, Tamblyn R (2021). Association of opioid consumption profiles after hospitalization with risk of adverse health care events. JAMA Netw Open.

[33] Chitnis SS, Tang R, Mariano ER (2020). The role of regional analgesia in personalized postoperative pain management. Korean J Anesthesiol.

[34] Nijs K, Ruette J, Van de Velde M, Stessel B (2023). Regional anaesthesia for ambulatory surgery. Best Pract Res Clin Anaesthesiol.

[35] Scott MJ (2024). Perioperative patients with hemodynamic instability: consensus recommendations of the anesthesia patient safety foundation. Anesth Analg.

[36] Khetarpal R, Chatrath V, Dhawan A, Attri JP (2016). Regional anesthesia in difficult airway: the quest for a solution continues. Anesth Essays Res.

